# Review of “Managed Care in a Public Setting”

**DOI:** 10.3389/fpubh.2014.00092

**Published:** 2014-07-23

**Authors:** David O. Carpenter

**Affiliations:** ^1^Institute for Health and the Environment, University at Albany, The State University of New York, Rensselaer, NY, USA

**Keywords:** community-based programs, managed care programs, health care delivery, health care reform, decentralized control

Rick Steele makes a passionate case for a more community-based system of managed health care with the overall goal of decentralization of services and the expectation that this will result in lower costs without lowering of services.

Drawing on his long and rich experiences as a practicing physician in Denmark and with public health training in the United States, Rick Steele has a broad perspective on the various forms used for delivery of health care in both Europe and the United States. He finds both positives and inadequacies in the delivery of health care in both continents and makes specific proposals for the development of a managed care system which is responsive to local needs and consists of an amalgamation of health and social services. This proposes that much of managed care delivery be done at the local level, and that this can be accomplished if communities set priorities, work to coordinate service, and promote inter-sector cooperation. He repeatedly emphasizes the problems that result when medical care is not adequately coordinated with social services. The result of failure to integrate health and social services delivery is both increased costs and reduced patient satisfactory and care.

Steele provides chapters on staffing and development of a model program with specific suggestions for implementation and evaluation. These are presented with a clear understanding that an optimal system must be responsive to local needs, which will not be the same in every community.

This book is a must read for anyone who is concerned about improving health care while at the same time reducing costs.

## Conflict of Interest Statement

The author declares that the research was conducted in the absence of any commercial or financial relationships that could be construed as a potential conflict of interest.

